# Impact of prostate cancer testing: an evaluation of the emotional consequences of a negative biopsy result

**DOI:** 10.1038/sj.bjc.6605648

**Published:** 2010-04-06

**Authors:** R C Macefield, C Metcalfe, J A Lane, J L Donovan, K N L Avery, J M Blazeby, L Down, D E Neal, F C Hamdy, K Vedhara

**Affiliations:** 1 Department of Social Medicine, University of Bristol, Canynge Hall, 39 Whatley Road, Bristol BS8 2PS, UK; 2 University Department of Oncology, Addenbrooke's Hospital, Hills Road, Cambridge CB2 0QQ, UK; 3 Nuffield Department of Surgery, University of Oxford, John Radcliffe Hospital, Oxford OX3 9DU, UK; 4 I-WHO, University of Nottingham, International House, Jubilee Campus, Wollaton Road, Nottingham NG8 1BB, UK

**Keywords:** PSA testing, prostate biopsy, psychological distress, screening

## Abstract

**Background:**

When testing for prostate cancer, as many as 75% of men with a raised prostate-specific antigen (PSA) have a benign biopsy result. Little is known about the psychological effect of this result for these men.

**Methods:**

In all, 330 men participating in the prostate testing for cancer and treatment (ProtecT) study were studied; aged 50–69 years with a PSA level of ⩾3 ng ml^−1^ and a negative biopsy result. Distress and negative mood were measured at four time-points: two during diagnostic testing and two after a negative biopsy result.

**Results:**

The majority of men were not greatly affected by testing or a negative biopsy result. The impact on psychological health was highest at the time of the biopsy, with around 20% reporting high distress (33 out of 171) and tense/anxious moods (35 out of 180). Longitudinal analysis on 195 men showed a significant increase in distress at the time of the biopsy compared with levels at the PSA test (difference in Impact of Events Scale (IES) score: 9.47; 95% confidence interval (CI) (6.97, 12.12); *P*<0.001). These levels remained elevated immediately after the negative biopsy result (difference in score: 7.32; 95% CI (5.51, 9.52); *P*<0.001) and 12 weeks later (difference in score: 2.42; 95% CI (0.50, 1.15); *P*=0.009). Psychological mood at the time of PSA testing predicted high levels of distress and anxiety at subsequent time-points.

**Conclusions:**

Most men coped well with the testing process, although a minority experienced elevated distress at the time of biopsy and after a negative result. Men should be informed of the risk of distress relating to diagnostic uncertainty before they consent to PSA testing.

Prostate cancer is a serious public health problem, motivating research to determine whether population screening is effective. The recent publication of two randomised controlled trials still leaves the benefits of screening uncertain and controversial (Andriole *et al*, 2009; Schröder *et al*, 2009), with the European Association of Urology currently not recommending screening as a public health policy (Abrahamsson *et al*, 2009). Regardless of the lack of evidence, prostate-specific antigen (PSA) testing in asymptomatic men continues to rise (Melia *et al*, 2004; Collin *et al*, 2008).

Uncertainties remain about the predictive validity of PSA tests, and their ability to identify tumours that will progress to cause morbidity and mortality (Frankel *et al*, 2003; Holmstrom *et al*, 2009). Arbitrary thresholds (commonly 3 or 4 ng ml^−1^) are used to recommend referral for biopsy. PSA levels are affected by measurement error and conditions other than prostate cancer, so it is not uncommon for PSA levels to rise and fall (Rosario *et al*, 2008). For these reasons, a high proportion of men with a raised PSA go on to receive a negative biopsy result – for example, 75% of men with a PSA test of ⩾3 ng ml^−1^ who had a biopsy in the European Randomised Study of Screening for Prostate Cancer (Schröder *et al*, 2009).

Consequently, many men who have a PSA test (and their physicians) may be left to cope with uncertain results. Increases in negative mood have been found in studies of women with abnormal but benign results for breast (Lowe *et al*, 1999; Aro *et al*, 2000) and ovarian (Andrykowski *et al*, 2004) cancer. Although studies of men undergoing PSA testing have shown no significant effect on anxiety for those receiving an abnormal PSA result (Essink-Bot *et al*, 1998; Brindle *et al*, 2006; Carlsson *et al*, 2007), it has been reported that those who receive a benign biopsy have thought and worried more about prostate cancer (McNaughton-Collins *et al*, 2004; Fowler *et al*, 2006; Katz *et al*, 2007). However, these latter studies were small and relied on unvalidated measures for these outcomes. We aimed to assess the prevalence and level of psychological distress and negative mood at several time-points during population-based testing for prostate cancer, focusing on men who received a negative biopsy result, using validated and standardised questionnaires. Baseline data were explored to investigate whether men vulnerable to heightened distress could be identified early in the testing process.

## Materials and methods

### Participants

Participants in this study were men enrolled in the Prostate testing for cancer and Treatment (ProtecT) study, a randomised trial of treatment for localised prostate cancer (Donovan *et al*, 2003). Men aged 50–69 years were invited from general practices across nine sites in the United Kingdom to attend for PSA testing. Those with a raised PSA level (⩾3 ng ml^−1^) were offered a transrectal ultrasound-guided biopsy carried out by an urologist to a standard 10-core protocol at the local hospital. Men diagnosed with clinically localised cancer were eligible for randomisation to one of the three treatments. Between June 2007 and September 2008, men receiving a negative biopsy result (with no immediate requirement for a re-biopsy) were identified for this substudy, by local research nurses from eight of the UK sites.

Approval for the ProtecT study was obtained from Trent Multi-centre Research Ethics Committee. Written informed consent was provided by all participants.

### Measures

**Figure 1:**
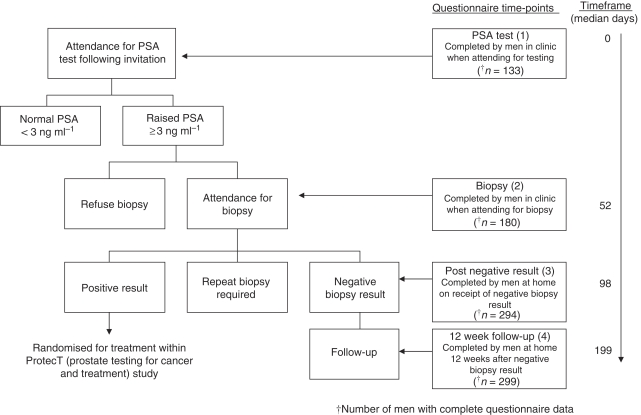
Outline of study design and assessment time-points.

Mood and psychological distress were assessed at four time-points by patient-completed questionnaires: (1) when attending for the first PSA test (before the result was known); (2) when attending for biopsy (in clinic before the procedure); (3) within a few days of receiving the negative biopsy result (postal questionnaire, completed at home) and (4) ∼12 weeks later (postal questionnaire, for those who returned a questionnaire at time-point 3) (see Figure 1). One reminder was posted if questionnaires were not returned within 10 working days.

Current states of mood were assessed by the Profile of Mood States – short form (POMS-SF) (Shacham, 1983), a 37-adjective checklist rated on a 5-point scale (0=‘not at all’ to 4=‘extremely’). Responses to items were totalled, providing six subscale scores for tension-anxiety (maximum score=24), depression-dejection (maximum score=32), anger-hostility (maximum score=28), fatigue-inertia (maximum score=20), vigour-activity (maximum score=24) and confusion-bewilderment (maximum score=20). Distress was measured by the Impact of Events Scale (IES) (Horowitz *et al*, 1979); in which seven items formed an intrusion subscale and eight an avoidance subscale, scored 0=‘not at all’, 1=‘rarely’, 3=‘sometimes’, 5=‘often’, and collated to give an overall score (maximum score=75). Questions were adapted to assess the frequency of intrusive thoughts and avoidance of issues surrounding the specific event of that time-point: the PSA test, the biopsy, the negative biopsy result and, for the follow-up assessment, being tested for prostate cancer overall. For example: ‘I tried not to think about the PSA blood test’ (time-point 1); ‘I tried not to think about going for my biopsy’ (time-point 2); ‘I tried not to think about getting my biopsy results’ (time-point 3) and ‘I tried not to think about being tested for prostate cancer’ (time-point 4). Written instructions informed men to respond about their feelings during the past week, including that day. Both measures have previously been used in studies investigating the effect of cancer screening (Taylor *et al*, 2002; Andrykowski *et al*, 2004).

### Missing data

Multiple responses to an individual item on the POMS questionnaire were considered as errors and treated as missing values. Single missing values within a subscale were replaced with the individual's mean response to items within the rest of that subscale (184 imputations, <1% of total responses). In cases of two or more missing values, no score was calculated for that POMS subscale. Missing items on the IES resulted in no subscale score or total score. This substudy was embedded in the ProtecT trial and as the POMS and IES were added in an amendment to the original protocol, some men identified with a negative biopsy result had completed time-points 1 and 2 before the measures were introduced, resulting in lower numbers of men completing the measures at earlier time-points (see Figure 1).

### Statistical analyses

High levels of psychological distress were determined using established cutoff thresholds: for IES, a total score of >19 (Joseph, 2000); for POMS negative mood subscales, 1.5 s.d. above the mean at PSA testing (time-point 1) (Nyenhuis *et al*, 1999). Change in mood over time was examined in those men who completed all of the questionnaires they were sent (*n*=195); this included men who had already undergone PSA testing and biopsy when this substudy started and so had their earlier questionnaire responses missing completely at random. *T*-tests compared the scores at PSA test for this cohort of men to those who completed questionnaires at PSA test but not all of the subsequent assessments. For longitudinal analyses, linear regression models with questionnaire subscale scores as outcome measures were fitted using Stata 10. Models compared questionnaire scores at the subsequent time-points to responses at PSA test (time-point 1). *P*-values were calculated using parametric bootstrap estimates of the s.e. (Davison and Hinkley, 1997). Confidence intervals (CIs) were calculated using the percentile bias corrected and accelerated bootstrap method. A total of 1999 bootstrap samples were obtained by re-sampling men from the study sample with replacement, so accommodating the repeated measures design and any non-normality in the distributions of outcome measure. Because of the extreme positive skew of IES scores, a sensitivity analysis used logistic regression of dichotomised scores (no distress symptoms/at least some symptoms).

A secondary analysis examined cases of heightened distress (IES total score) and high anxiety (POMS tension-anxiety score) at attendance for biopsy and after having received a negative biopsy result, and explored potential predictors: demographic (age, family history of prostate cancer and any cancer), clinical (PSA level, lower urinary tract symptoms: frequency, urgency, incontinence, nocturia, hesitancy and interference with everyday life) and psychological (baseline POMS anxiety and IES avoidance and intrusion subscale scores). Univariable logistic regression models were fitted to examine each of these 13 baseline measures in turn as a predictor of high distress and anxiety at later time-points in the testing process.

## Results

In total, 330 men were contacted after a negative biopsy result. Table 1 shows their clinical and demographic details. The response rate for postal questionnaires was 91.8% after the negative biopsy result (time-point 3) and 82.6% at the 12-week follow-up (time-point 4). Non-responders were significantly younger than responders at both time-points (*P*=0.016, *P*=0.006, respectively). After accounting for non-responders and missing responses on questionnaire items, data were available from 294 out of 330 and 229 out of 287 men, respectively (only those who responded at time-point 3 were sent a follow-up questionnaire, and during one week men were not posted questionnaires due to an administrative error, thus reducing the denominator to 287). POMS and IES data were available for 133 out of 330 of the study sample at PSA assessment (time-point 1), and for 180 out of 330 at biopsy assessment (time-point 2) – smaller numbers due to introducing the measures part-way through the ProtecT study. Figure 1 shows average time (median number of days) between assessments.

Overall, rates of psychological distress (IES scores) and negative mood (POMS scores) were relatively low at all time-points, with around 80–95% of individuals reporting levels below the clinical threshold at each stage (Table 2). However, nearly one fifth of men (19.4%) reported high levels of tension-anxiety at the time of attending for the biopsy (time-point 2), and 8.9% after the negative biopsy result (time-point 3). The proportion of men with a distress level of clinical concern was markedly higher at the time of biopsy compared with distress at the time of the PSA test (19.3, 0.8%, respectively). This percentage decreased only slightly after the negative biopsy result (16.9%), and 9.7% were distinctly distressed by the testing process after 12 weeks. Reports of high depression-dejection (7.5%), anger-hostility (6.8%), fatigue-inertia (12%) and confusion-bewilderment (8.3%) were most prevalent at the time of the PSA test, whereas they were <5% by the 12-week follow-up (Table 2).

Within the study sample, 195 men responded to all questionnaires they were sent, with POMS and IES scores missing for one or both of the first two assessments only if the man joined the study before these measures were introduced. These earlier assessments at time-points 1 and 2 were assumed to be missing at random, enabling changes in mood over the course of the testing process to be observed without confounding by determinants of non-response (Table 3). This cohort had similar clinical and demographic distributions to that of the complete sample (Table 1). At the time of the biopsy, a significant increase in tense and anxious moods was apparent (difference in mean: 2.07; 95% CI (1.35, 2.83); *P*<0.001), and men experienced significantly more distress (difference in mean IES total score: 9.47; 95% CI (6.97, 12.12); *P*<0.001). After receiving a negative biopsy result, scores for tense and anxious moods returned to levels similar to those recorded at the PSA test. However, reports of distress were significantly higher than at the time of the PSA test (difference in mean: 7.32; 95% CI (5.51, 9.52); *P*<0.001). Approximately 12 weeks after having received a negative result (time-point 4), distress relating to prostate cancer testing remained more prevalent than distress at the time of the PSA test (difference in mean: 2.42; 95% CI (0.50, 1.15); *P*=0.009), with 8.9% of men (16 out of 179) reporting scores of clinical concern. For these IES results, logistic regression of dichotomised scores supported the same conclusions, although this sensitivity analysis suggested that the elevation at the 12-week follow-up assessment could have arisen by chance (odds ratio (OR): 1.42; 95% CI (0.80, 2.52); *P*=0.23). Feelings of confusion-bewilderment were at a significantly lower level at the 12-week follow-up than at PSA testing (difference in mean: −0.73; 95% CI (−1.26, −0.25); *P*=0.004). Additional analysis of the cohort of men with complete data available for all four of the assessment time-points (*n*=66) was consistent with the results presented here and the same conclusions are supported.

None of the demographic or clinical factors recorded at baseline significantly predicted high distress and anxiety levels at the subsequent study time-points (with the exception of urinary hesitancy: OR: 4.76; 95% CI (1.95, 11.62); *P*=0.001), although psychological factors did (Table 4).

For *post hoc* analysis to explore other possible reasons why men may experience higher distress levels 12 weeks after biopsy, we retrieved information on any further biopsies the study men had undergone. Twenty-three men were found to have had a second biopsy before being sent their 12-week follow-up questionnaires, of which 17 men returned data for this assessment (73.9%). The mean IES total scores (s.d., number of men with high scores/number completing measure) for these 17 men were 1.29 (2.98, 0 out of 7) at time-point 1; 5.00 (4.38, 0 out of 6) at time-point 2; 9.12 (10.75, 3 out of 17) at time-point 3 and 8.53 (11.31, 4 out of 15) at time-point 4. There was no significant difference between IES scores at the 12-week follow-up assessment for those who had a repeat biopsy and those who did not (*P*=0.096). Of the total number of men from the complete cohort who had high distress at the 12-week follow-up assessment, 18% (4 out of 22) had undergone a further biopsy.

## Discussion

This study shows that most men seem to cope well with a raised PSA result followed by a negative biopsy. Mean scores for all the questionnaire scales were relatively low with regard to the thresholds for clinical significance and the maximum possible ranges. However, nearly 20% experienced high levels of tension-anxiety and psychological distress at the time of the biopsy, as well as 8.9% with high tension-anxiety and 16.9% with psychological distress immediately after the receipt of a negative biopsy result. These findings were substantiated in the longitudinal cohort. Men exhibited significant increases in distress and tense and anxious moods from baseline to the time of the biopsy, which then declined somewhat after receipt of a negative biopsy result, before returning to much lower levels 12 weeks later. However, distress was significantly more prevalent at all of the subsequent time-points compared with distress at the time of the PSA test, and almost 10% reported distress levels of clinical concern at the 12-week follow-up. Baseline levels of mood were found to predict levels of distress and anxiety at the time of biopsy, after the negative biopsy result and 12 weeks later.

The present investigation was conducted within the ProtecT study, enabling large numbers of men based in the community to participate. This detailed quantitative study of the emotional consequences of prostate cancer testing is the first study of its kind in the United Kingdom, using more detailed psychological measures and using a longer follow-up time than one other study conducted in the Netherlands (Essink-Bot *et al*, 1998). However, the study encompasses some limitations. The ‘baseline’ measures were at the time of the initial PSA test, and it could be argued that negative mood may already have been elevated at this time, although an earlier investigation in the ProtecT cohort showed no difference in depressed or anxious mood between men who responded to an invitation for PSA testing and those who did not (Avery *et al*, 2008b). All men had self-selected to take part in the study initially, which may influence their psychological response to being tested. An important issue when interpreting the findings from the longitudinal cohort was that they were a smaller and select group of men. This had the advantage of exploring unconfounded comparisons across time, although external validity was weakened as men who failed to respond were excluded. Caution is therefore needed before generalising these results to a wider population. The statistical methodology chosen did, however, make use of the maximum data available. The longitudinal cohort included those men who had already undergone their biopsy just before the current substudy started, but who completed both of their post-biopsy questionnaires when invited to do so. These men did not complete substudy questionnaires at time-points 1 and 2, but as these data are missing completely at random, their inclusion in the longitudinal cohort improves precision without introducing bias. Nevertheless, we acknowledge that these results could have been strengthened by a longer recruitment period enabling a larger longitudinal cohort with data available at all four assessments.

The study findings are reassuring in that most men seemed to cope well with the seemingly equivocal result of a raised PSA followed by a negative biopsy result. However, it remains of concern that nearly 20% suffered high levels of distress at the time of biopsy and nearly 17% after having received a negative result. In an earlier longitudinal study of men being screened for prostate cancer, Gustafsson *et al* (1995) reported that cortisol level (indicating the degree of emotional stress) was highest immediately before being informed of the biopsy result, although levels decreased somewhat 2 weeks later in those who received a benign biopsy result. Other studies have concluded that testing for prostate cancer had little or no effect on men's psychological health (Brindle *et al*, 2006; Awsare *et al*, 2008). These studies relied on the Hospital Anxiety and Depression Scale (HADS) (Zigmond and Snaith, 1983), which was designed to detect clinical cases of anxiety and depression. Both studies commented on the low sensitivity of the HADS in the context of prostate cancer testing. In addition, a longitudinal Dutch screening study reported no significant adverse effects on psychological health after receipt of a negative biopsy result (Essink-Bot *et al*, 1998). This study used a general measure for anxiety, which also may not have been sensitive enough to identify men's concerns in the context of prostate cancer screening. The findings reported here confirm that more detailed, specific measures (e.g. POMS and IES) better reflect the changes in mood and distress in some men as apparent in an earlier qualitative study (Avery *et al*, 2008a).

The IES statements at each of the first three time-points were tailored to focus on specific aspects current for that stage of the testing process, for example at time-point 1: ‘I had dreams about the PSA blood test’. The adapted IES statements at the 12-week follow-up (time-point 4) referred to the complete testing process for example ‘I had dreams about being tested for prostate cancer’. In contrast, the POMS consisted of an adjective list of emotions with no direct reference to particular events in time. The pattern of mood levels over time was similar whether measured by the IES or POMS, although POMS scores seemed to return to pre-PSA levels sooner. At every time-point, men were asked to respond with regard to how they had felt during the preceding week. However, at the 12-week follow-up, the prompt to think about the complete testing process may have triggered memories of earlier mood states. This could have influenced men's responses, rather than just reporting on recent intrusive thoughts and avoidant behaviours that had troubled them during the previous week. It is possible that distress in some men 12 weeks after the biopsy could be due to the knowledge that a negative biopsy result does not necessarily indicate the ‘all clear’. Further research, preferably qualitative, is required to explore this issue and other explanations for distress after a negative biopsy result.

It has previously been shown that older age, a positive family history, and a higher PSA level did not predict anxiety in men during testing for prostate cancer (Macefield *et al*, 2009). In contrast here, baseline psychological mood was predictive of distress and anxiety at later stages of the testing process. This reflects the findings of the Dutch study, where initial high anxiety levels were maintained during testing (Essink-Bot *et al*, 1998), and supports that baseline psychological factors can continue to be predictive of distress and anxiety after a longer period after screening.

The findings of this study have clear practical relevance. Despite its controversy, PSA testing is widespread. These results, particularly that high levels of distress may be encountered by some men, should be included in information presented to men by GPs before they consent to receiving a test. For men undergoing testing, whether as part of screening, or in primary or secondary care, the concomitant collection of POMS and IES data could be used to identify men who are experiencing tension-anxiety and early distress symptoms at the time of the PSA test, and thus identify those who might benefit from additional support and information to prevent this developing into distress later on (see also Essink-Bot *et al*, 1998; Awsare *et al*, 2008).

The intention of this study was to capture men's psychological experiences during prostate cancer testing. Focusing on those who received a negative biopsy result, it revealed that the majority of men who undergo testing are not significantly adversely affected, although some men do find the process distressing. Men should be informed of the risk of distress before agreeing to a PSA test.

## Figures and Tables

**Table 1:** Clinical and demographic information for the whole study sample and the longitudinal cohort

	**Whole study sample (*n*=330)**		**Longitudinal cohort (*n*=195)**	
	**Summary statistic**	**Range**	**Summary statistic**	**Range**
Mean age, years (s.d.)	62.3 (4.58)	50.5–70.6	62.7 (4.52)	51.3–70.6
Mean PSA level at first test, ng ml^−1^ (s.d.)	4.6 (1.9)	3.0–17.0^a^	4.6 (1.8)	3.0–15.2^a^
% Family history of any cancer (*n*)	53.6 (177)	—	51.8 (101)	—
% Family history of prostate cancer (*n*)	8.2 (27)	—	8.7 (17)	—
% White ethnicity (*n*)	95.5 (315)	—	95.9 (187)	—
% Undergoing first biopsy (*n*)	93.6 (309)	—	93.3 (182)	—

Abbreviations: ProtecT=prostate testing for cancer and treatment; PSA=prostate-specific antigen.

a Men with PSA⩾20 ng ml^−1^ are excluded from ProtecT study.

*n*=number in sample.

**Table 2:** Mean scores for negative moods and distress, and percentage of men with scores of clinical significance^a^

	**PSA test (time-point 1)**		**Biopsy (time-point 2)**		**Post-negative result (time-point 3)**		**12-week follow-up (time-point 4)**	
**Questionnaire and subscale**	**Mean (s.d.)**	**% High scores (*n*/*N*)**	**Mean (s.d.)**	**% High scores (*n*/*N*)**	**Mean (s.d.)**	**% High scores (*n*/*N*)**	**Mean (s.d.)**	**% High scores (*n*/*N*)**
POMS tension-anxiety	3.77 (3.48)	10.5 (14/133)	5.07 (4.11)	19.4 (35/180)	3.38 (3.75)	8.9 (26/292)	2.39 (2.89)	4.8 (11/229)
Depression-dejection	2.52 (4.57)	7.5 (10/133)	2.42 (3.65)	5.1 (9/178)	2.40 (4.10)	6.5 (19/292)	1.75 (3.49)	3.1 (7/228)
Anger-hostility	2.94 (3.56)	6.8 (9/132)	2.89 (3.37)	5.6 (10/179)	2.40 (3.43)	6.2 (18/292)	1.89 (2.68)	3.9 (9/229)
Fatigue-inertia	4.44 (4.30)	12.0 (16/133)	3.34 (3.37)	5.0 (9/180)	3.83 (4.19)	7.5 (22/294)	3.44 (3.53)	4.4 (10/229)
Confusion-bewilderment	2.62 (2.67)	8.3 (11/133)	2.56 (2.56)	6.7 (12/178)	2.16 (2.66)	6.9 (20/292)	1.75 (2.19)	4.8 (11/229)
IES distress (total score)	2.35 (4.43)	0.8 (1/129)	11.74 (13.76)	19.3 (33/171)	9.51 (12.32)	16.9 (49/290)	4.88 (8.82)	9.7 (22/227)

Abbreviations: IES=Impact of Events Scale; POMS=Profile of Mood States; PSA=prostate-specific antigen.

a Thresholds for clinical scores (see text for definitions): POMS tension-anxiety 9.0, depression-dejection 9.4, anger-hostility 8.3, fatigue-inertia 10.9, confusion-bewilderment 6.6; IES distress 19.0.

*n*/*N*=number of cases/number completing subscale.

Data are cross-sectional at each assessment time-point, from the whole study sample.

**Table 3:** Mean scores for the longitudinal cohort (those who completed all the questionnaires they were sent)

	**PSA test (time-point 1)**		**Biopsy (time-point 2)**		**Post-negative result (time-point 3)**		**12-week follow-up (time-point 4)**		
**Questionnaire and subscale**	**Mean (s.d.)**	**% High scores (*n*/*N*)**	**Mean (s.d.)**	**% High scores (*n*/*N*)**	**Mean (s.d.)**	**% High scores (*n*/*N*)**	**Mean (s.d.)**	**% High scores (*n*/*N*)**	***P*-value^a^**
POMS tension-anxiety	2.95 (2.83)	4.6 (3/66)	5.02 (4.31)	19.1 (25/131)	3.17 (3.66)	8.8 (17/194)	2.31 (2.77)	3.6 (7/194)	<0.001
Depression-dejection	1.72 (2.57)	1.5 (1/65)	2.38 (3.65)	5.5 (7/128)	1.99 (3.62)	5.2 (10/191)	1.60 (3.07)	2.1 (4/191)	0.086
Anger-hostility	2.37 (2.56)	4.6 (3/66)	2.71 (3.31)	5.4 (7/130)	2.18 (3.39)	5.7 (11/194)	1.88 (2.61)	3.6 (7/194)	0.041
Fatigue-inertia	3.42 (3.22)	3.0 (2/66)	3.17 (3.38)	4.6 (6/131)	3.22 (3.56)	4.1 (8/195)	3.24 (3.34)	3.1 (6/195)	0.95
Confusion-bewilderment	2.41 (2.17)	6.1 (4/66)	2.43 (2.49)	7.0 (9/129)	1.88 (2.29)	3.6 (7/193)	1.68 (2.07)	4.2 (8/193)	0.002
IES distress (total score)	2.52 (5.20)	1.7 (1/60)	11.98 (14.23)	21.1 (24/114)	9.84 (12.74)	19.0 (34/179)	4.93 (9.01)	8.9 (16/179)	<0.001

Abbreviations: IES=Impact of Events Scale; POMS=Profile of Mood States; PSA=prostate-specific antigen.

a Overall test of differences across all four means.

*n*/*N*=number of cases/number in sample.

**Table 4:** Predictor variables for high anxiety and psychological distress at biopsy, after receiving a negative biopsy result, and 12 weeks later

		**Anxiety^a^**		**Distress^b^**			
**Predictor**	**Time-point**	***N***	**OR (CI)**	***P*-value**	***N***	**OR (CI)**	***P*-value**
Baseline^c^ tense/anxious mood	(2) Biopsy	90	1.34 (1.12–1.61)	0.001	86	1.13 (0.98–1.30)	0.09
	(3) Post-negative result	122	1.37 (1.16–1.62)	<0.001	117	1.03 (0.91–1.16)	0.65
	(4) After 12 weeks	93	1.55 (1.13–2.13)	0.007	93	1.16 (0.95–1.43)	0.15
							
Baseline^c^ intrusive thoughts	(2) Biopsy	90	1.53 (1.16–2.01)	0.002	87	1.43 (1.11–1.85)	0.006
	(3) Post-negative result	122	1.43 (1.17–1.74)	0.001	119	1.17 (1.00–1.36)	0.049
	(4) After 12 weeks	94	1.21 (0.98–1.48)	0.07	96	1.21 (1.00–1.45)	0.047
							
							
Baseline^c^ avoidant behaviours	(2) Biopsy	85	1.29 (1.07–1.55)	0.008	82	1.23 (1.03–1.48)	0.02
	(3) Post-negative result	115	1.31 (1.09–1.57)	0.004	113	1.29 (1.06–1.57)	0.01
	(4) After 12 weeks	91	1.06 (0.79–1.42)	0.71	93	1.08 (0.86–1.36)	0.50

Abbreviations: CI=95% confidence interval; OR=odds ratio; PSA=prostate-specific antigen.

a Based on Profile of Mood State tension-anxiety score.

b Based on Impact of Events Scale total score.

c Baseline mood and distress is that measured at time of initial PSA testing.
